# Towards Transabdominal Functional Photoacoustic Imaging of the Placenta: Improvement in Imaging Depth Through Optimization of Light Delivery

**DOI:** 10.1007/s10439-021-02777-0

**Published:** 2021-04-28

**Authors:** Kristie Huda, Kenneth F. Swan, Cecilia T. Gambala, Gabriella C. Pridjian, Carolyn L. Bayer

**Affiliations:** 1 Department of Biomedical Engineering, Tulane University, 500 Lindy Boggs Center, New Orleans, LA 70118 USA; 2 Department of Obstetrics and Gynecology, Tulane University, 1430 Tulane Ave, New Orleans, LA 70112 USA; 3 School of Medicine, Tulane University, 1430 Tulane Ave, New Orleans, LA 70112 USA

**Keywords:** Photoacoustic imaging, Placenta, Monte Carlo simulation, Transabdominal imaging

## Abstract

Functional photoacoustic imaging of the placenta could provide an innovative tool to diagnose preeclampsia, monitor fetal growth restriction, and determine the developmental impacts of gestational diabetes. However, transabdominal photoacoustic imaging is limited in imaging depth due to the tissue’s scattering and absorption of light. The aim of this paper was to investigate the impact of geometry and wavelength on transabdominal light delivery. Our methods included the development of a multilayer model of the abdominal tissue and simulation of the light propagation using Monte Carlo methods. A bifurcated light source with varying incident angle of light, distance between light beams, and beam area was simulated to analyze the effect of light delivery geometry on the fluence distribution at depth. The impact of wavelength and the effects of variable thicknesses of adipose tissue and muscle were also studied. Our results showed that the beam area plays a major role in improving the delivery of light to deep tissue, in comparison to light incidence angle or distance between the bifurcated fibers. Longer wavelengths, with incident fluence at the maximum permissible exposure limit, also increases fluence within deeper tissue. We validated our simulations using a commercially available light delivery system and *ex vivo* human placental tissue. Additionally, we compared our optimized light delivery to a commercially available light delivery system, and conclude that our optimized geometry could improve imaging depth more than 1.6×, bringing the imaging depth to within the needed range for transabdominal imaging of the human placenta.

**Electronic supplementary material:**

The online version of this article (10.1007/s10439-021-02777-0) contains supplementary material, which is available to authorized users.

## Introduction

The placenta is a vital organ that develops during pregnancy to support fetal growth and viability, providing oxygen, transport, and metabolism of nutrients, as well as endocrine and immunity functions.^19^ Abnormal placental function can lead to fetal growth restriction,^8^ and is a clinical presentation of both gestational diabetes,^10^ and preeclampsia.^17^ Abnormal placental function may be a result of either the improper remodeling of the uterine spiral arteries or insufficient decidual invasion, leading to abnormal uteroplacental circulation.^9^ This abnormal uteroplacental circulation can lower oxygenation of the placenta due to reduced placental perfusion and blood volume.^38^ Standard ultrasound can image anatomy (e.g. placenta thickness, volume, echogenicity, location, etc.) but not function. Doppler ultrasound provides measures of uterine spiral artery blood flow, which are secondary indicators of function, as demonstrated in patients with preeclampsia and fetal growth restriction.^33^,^35^,^42^ However, since the maternal health and cardiovascular system physiology also affect uterine artery spiral blood flow, measures of blood flow are limited in diagnostic capability.^15^,^46^ BOLD MRI can measure changes in placental perfusion or oxygenation by exploiting the interference of deoxyhemoglobin on the magnetic field which results in reduced signal intensity.^18^ However, quantitative assessment of oxygenation using BOLD MRI is hampered by the dependence of the signal on other physiological parameters of the placenta.^32^

Spectral photoacoustic imaging could potentially measure placental oxygenation directly, which would be beneficial for diagnostics during pregnancy. Photoacoustic imaging combines the benefits of both optical and ultrasound imaging to produce high-contrast images of tissue composition and function.^6^,^13^,^44^,^48^ In photoacoustic imaging, nonionizing nanosecond pulses of light excite chromophores in the tissue, such as hemoglobin. Absorption of the light by the tissue chromophores results in localized heating. This transient heating, and expansion, followed by cooling and contraction, generates acoustic waves within the tissue which can be detected using a standard ultrasound transducer. Due to the wavelength-dependent optical properties of chromophores such as hemoglobin, oxyhemoglobin, lipid, melanin, and water, the distinction of unique chromophores is possible by varying the wavelength of the laser light source, using the photoacoustic signal intensity as a function of wavelength to distinguish the resulting signals.^12^,^14^ This approach, termed spectral photoacoustic imaging, has been demonstrated to monitor longitudinal placental oxygenation in a preeclamptic rat model.^28^ While photoacoustic imaging has shown promise in the detection of cancer,^29^,^31^ monitoring angiogenesis,^27^ and to detect cardiovascular disease,^24^,^45^ the clinical translation of photoacoustic imaging is limited by the challenge of delivering sufficient laser fluence at significant tissue depths.

The achievable imaging depth of photoacoustic imaging depends on the fluence deposited in the tissue of interest.^12^ Since the initial photoacoustic wave is proportional to the fluence within the tissue, if a higher fluence can be delivered to the location of interest, a higher photoacoustic signal can be generated. The design of the light delivery system can alter the local tissue fluence, and therefore change the maximum photoacoustic imaging depth.^20^,^21^,^37^,^39^,^41^,^43^,^49^ Early simulations of the delivery of light to biological tissues found that increasing the diameter of a circular beam area increases the depth of light delivery.^3^,^25^ However, the limited depth (< 0.5 cm) modeled in these simulations artificially produces a plateau in the optimal beam diameter, and therefore cannot be extrapolated to the delivery of light deeper within tissue. Additionally, these initial simulations only considered a single layer of tissue, and only considered a single geometric parameter (beam diameter). Specific to photoacoustic imaging, prior studies to assess the impact of beam length on photoacoustic imaging depth maintained a constant energy.^37^ Reducing the beam width increased the surface incident fluence and therefore increased the achievable imaging depth, but this approach provides limited insight on how increasing the beam area, with a constant fluence, would impact photoacoustic imaging depth. For photoacoustic imaging, a dark-field, or side illumination, configuration is often implemented due to ease of integration with a linear array transducer. Therefore, a variable in the light delivery design for photoacoustic imaging has been the distance from the skin illumination site to the transducer imaging field of view.^21^,^41^,^43^ In human subjects, considering a relatively shallow (0.8–1.5 cm) imaging depth, an optimal distance between the transducer and light beam was found to be 1.5 cm.^21^ While the photoacoustic imaging demonstrations in humans are groundbreaking in this study, our ability to extrapolate from this data is limited since human experimental subjects will have uncontrolled sources of variation, such as tissue composition and heterogenicity, which would affect the absolute value predicted. In simulations, the distance between beam and transducer has been studied in a single layer homogeneous tissue.^41^ However, tissue composition does significantly impact the optimal distance from the illumination and transducer,^43^ and therefore we sought to study a more realistic tissue model of the abdomen, which cannot be captured in a single layer model. Likewise, the effect of incident angle on imaging depth is well studied, but dependent on tissue composition.^20^,^41^,^43^,^49^ Models exceeding 2 layers in complexity, which specifically simulate deep transabdominal tissues, have not been simulated to our knowledge. Other simulations of photoacoustic light delivery design investigated the effect of focal length;^39^ however, given tissue scattering, the focal length is unlikely to impact deep tissue imaging and therefore was excluded from our simulations. Using longer wavelengths for photoacoustic signal generation can also lead to higher fluence with increasing depth, but this is also is highly dependent on tissue composition.^40^ Therefore, a more complex multilayer tissue model representing the abdominal tissue was composed to specifically model this imaging scenario, providing data necessary to optimize the design of a light delivery system for photoacoustic imaging of the human placenta.

Therefore, in this study, we sought to comprehensively analyze the impact of the light delivery geometry and wavelength, targeted towards the unique imaging environment of the placenta. We used four parameters to optimize the light delivery geometry: incident angle, distance between bifurcated light sources, beam width along the elevational axis of the US transducer, and beam length along the lateral axis of the US transducer. A four-layer tissue model was simulated, consisting of skin, subcutaneous adipose tissue, abdominal muscle, and placental tissue. We then simulated the optimized light delivery geometry with different light wavelengths at their MPE limit to investigate the effect of wavelength on the ability to deliver light deeper within tissue. Additionally, we investigated the effect of varying thicknesses of subcutaneous adipose tissue and abdominal muscle on our optimized light delivery design. Finally, we compared our simulation of the optimized light delivery design with the light delivery of a commercial photoacoustic imaging system to demonstrate the potential for improvement in photoacoustic imaging deeper within tissue. Our simulation study provides insights on strategies to optimize the design of the light delivery system for photoacoustic imaging, to improve imaging depth for *in vivo* transabdominal imaging.

## Materials and Methods

### Monte Carlo Simulations

Monte Carlo simulations were used to optimize the design of the light delivery for photoacoustic imaging of the placenta. An open-source software package, MCXLAB,^16^ was employed to simulate the photon propagation through a multilayer tissue model of the abdomen. The MCXLAB software package provides the flexibility to design the light source based on the dimension of the rectangular light source, incident angle, and position of the source in 3D coordinates with respect to the tissue volume. Further details of the simulation process are provided in the supplementary file (Sect. S1).

### Multilayer Tissue Model

**Figure 1:**
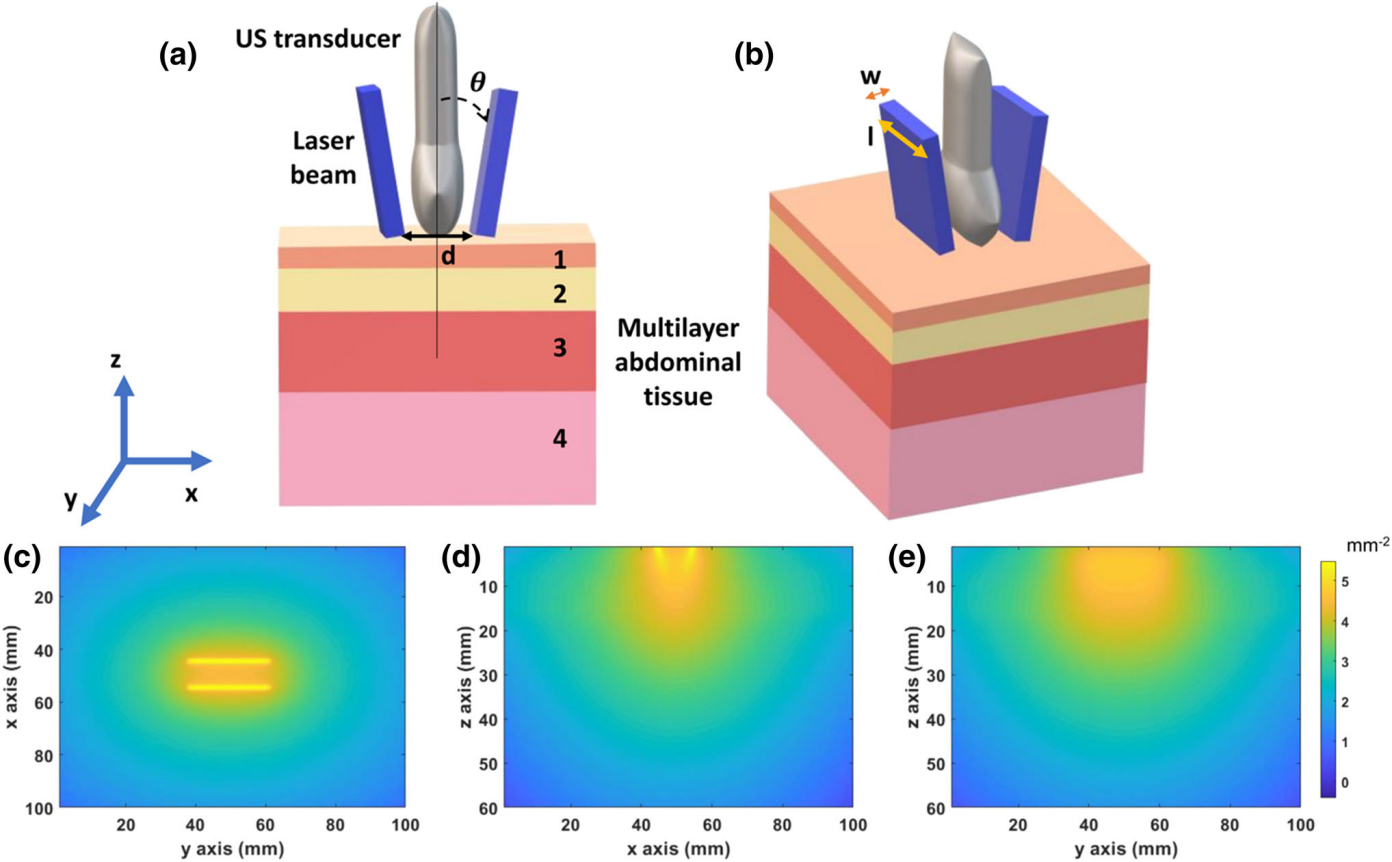
(**a**) Schematic diagram of the multilayer tissue model and the photoacoustic imaging transducer. The four layers are skin (1), subcutaneous adipose tissue (2), abdominal muscle (3), and placenta (4). The incident angle ($\varvec{\theta}$) and the distance (**d**) between the bifurcated light source is shown. (**b**) Laser beam width (w) along the elevational axis and beam length (**l**) are indicated. (**c**, **d**, **e**) 2D fluence maps of the bifurcated light source in the *xy*−, *xz*− and *yz*−planes, respectively.

A four-layered tissue model was used as a target for this simulation study. This multilayer tissue model contained skin, subcutaneous adipose tissue (SAT), abdominal muscle, and placenta. A schematic diagram of the multilayer tissue model is shown in Fig. 1(a). The total tissue volume was 100 × 100 × 60 mm with a voxel size of 1 mm^3^. We have chosen to model the placenta as a slab, as the third trimester placenta is large (~19 cm diameter)^4^ compared to the light beam area, and therefore it can be modeled as a semi-infinite medium.^34^ The thickness (t) of each tissue layer was estimated from literature, as shown in Table 1. The optical properties calculated for the simulation were the reduced scattering coefficient $\mu_{s}^{'}$, absorption coefficient $\mu_{a}$, anisotropy $g$ and refractive index $\eta$. The calculation of reduced scattering coefficient and absorption coefficient was performed using equations reported in the literature.^23^ The details of all calculations have been provided in the supplementary (Sect. S2). In our calculations, we used a 4.3% volume fraction of melanin so that our results may be compared to prior studies.^3^

**Table 1:** Multilayer tissue model parameters.

Tissue	$\mu_{a}$. (cm^−1^)				$\mu_{s}^{'}$. (cm^−1^)				$\eta$.	$g$.	*t* (mm)	References
	690 nm	808 nm	950 nm	1064 nm	690 nm	808 nm	950 nm	1064 nm				
Skin	9.68	5.65	3.5	2.28	21.5	18.04	5.10	13.3	1.4	0.9	2	1, 5, 7, 50
SAT	0.02	0.04	0.14	0.06	12.4	11.11	9.95	9.22	1.4	0.9	4	1, 5, 7, 50
Abdominal muscle	0.07	0.08	0.36	0.15	3.95	2.53	1.60	1.17	1.4	0.9	10	1, 7, 11, 50
Placenta	2.67	2.14	2.82	1.81	6.56	5.56	4.69	4.16	1.4	0.9	15	1, 7, 26, 50

The wavelengths of 690, 950, and 808 nm were selected for these simulations since they match peak absorptions of hemoglobin (Hb) and oxyhemoglobin (HbO_2_) as well as the isosbestic point of Hb and HbO_2,_ respectively. These wavelengths are often used for the generation of photoacoustic signal for imaging blood and to estimate tissue oxygen saturation.^30^ Additionally, we have simulated 1064 nm to determine the impacts of using light in the NIR-II in comparison to the NIR-I, since the allowable MPE is higher for the NIR-II.^47^ Specifically, within the NIR-II wavelength range we have chosen to simulate 1064 nm since it is a fundamental frequency of the Nd:YAG nanosecond lasers often used for photoacoustic signal generation from hemoglobin, and prior research has demonstrated experimentally that photoacoustic signal can be generated deeper within tissue at this wavelength.^22^,^47^ The absorption coefficients of each tissue at the wavelengths of interest are shown in Table 1. The refractive index $\eta$ and anisotropy $g$ were chosen to be 1.4 and 0.9 respectively for all tissues to avoid mismatch of refractive indices between layers.^5^,^23^ Since the optical properties of placental tissue are currently not reported in literature, we used the optical properties of the liver since it is an organ with a blood volume similar to the placenta.

### Modeling Light Delivery

We selected four parameters to investigate the impact of the geometry of the light delivery on the achievable fluence with increasing depth: incident angle of light beam normal to the surface of the skin (the vertical axis of US transducer) ($\theta$), distance between bifurcated light sources ($d$), beam width along the elevational axis of US transducer ($w$) and beam length along the lateral axis of US transducer ($l$). Darkfield illumination provided by a bifurcated light source, with one bifurcation on either side of a linear array ultrasound transducer, is a probe integration common for photoacoustic imaging systems, as it provides optimal alignment between the ultrasound and photoacoustic imaging planes. Additional geometric considerations in the design of the light delivery would comprehensively include those parameters mentioned above, as well as the distance from the light source to the tissue surface. Preliminary simulations, not shown here, indicated that the distance from the light source to the tissue surface had minimal impact on light delivery, but all other geometrical variables did have an impact and were included in the simulations shown here. Also, in our preliminary simulations, the effect of a non-planar or focused beam was found to be negligible since tissue scattering dominates to create a homogeneous beam within the superficial layers of tissue.

A schematic diagram of the light delivery geometry with these parameters is shown in Figs. 1(a) and 1(b). The bifurcated light source was directly incident on the surface of the skin of the multilayer tissue model with 1 mm space with a water layer between the light source and skin surface. To simulate the bifurcated light source, each incident light beam was simulated separately, and then the output fluence volumes were summed to get the final output fluence volume. The calculation of input energy of light source is described in detail in the supplementary (Sect. S3). We calculated the total fluence delivered to a 23 mm $\times$. 60 mm imaging plane shown in Fig. 1(e), matching the imaging plane of the LZ250 integrated photoacoustic and ultrasound imaging transducer (FUJIFILM VisualSonics, Inc., Toronto, Canada).

In our simulation geometry, the incident angle (*θ*) was varied, maintaining distance and beam area constant. Each light beam was positioned on the surface of the multilayer tissue model at *d*/2 distance from the vertical axis of the transducer. Distance was varied with constant incident angle and beam area for simulation. The width ($w$.) and length ($l$.) of the light beam indicated the elevational and lateral axes of the transducer, respectively were varied separately while maintaining constant incident angle and distance. The details of how we varied each parameter for the simulation are further described in the supplementary (Sect. S4).

We also varied the thickness of the subcutaneous adipose tissue and muscle layers in our simulations. Human subcutaneous adipose tissue thickness varies between 1 and 6 mm depending on body site,^5^ whereas abdominal muscle (rectus abdominis) thickness is 9.77±1.62 mm for non-pregnant women.^11^ During pregnancy, the rectus abdominis stretches and become thinner with gestational week, therefore, these non-pregnant estimates are likely a conservative estimate of the actual tissue thicknesses through which light must be delivered for photoacoustic imaging.

### *Ex Vivo* Tissue Imaging

To test whether our simulated light delivery system matches experimental data, we performed photoacoustic imaging of *ex vivo* placentas. Three term human placentas were collected after cesarean section from consented patients, following a protocol approved by Tulane University Institutional Review Board. The details of placental tissue preparation and photoacoustic imaging have been included in the supplementary (Sect. S5).

The raw photoacoustic images of each placental cotyledon were processed in MATLAB (Mathworks, Natick, MA, USA). To find the maximum photoacoustic imaging depth, first, the photoacoustic signal intensity along the *y* axis was summed at each *z* point of each image, normalized by the peak photoacoustic signal intensity. The average photoacoustic signal generated within the gel coupling layer was used as a noise threshold. The depth at which the photoacoustic signal fell below this noise threshold was defined as the maximum photoacoustic imaging depth.

## Results

### Monte Carlo Simulation Experiments

**Figure 2:**
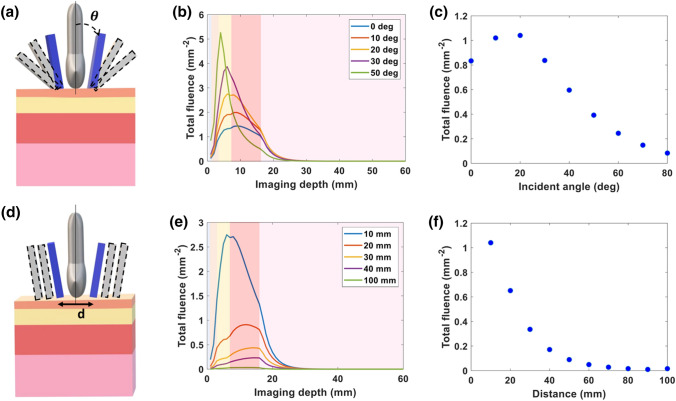
Impact of incident angle and distance between bifurcated fibers on the delivery of 808 nm light. (**a**) Schematic indicating the varying incident angle of light beam along the vertical axis of transducer. (**b**) Plot of total fluence versus imaging depth at varying incident angles. (**c**) Plot of total fluence at the surface of placenta (depth, *z* = 17 mm) versus incident angle. (**d**) Schematic indicating varying the distance between bifurcated light beam from the vertical axis of transducer. (**e**) Plot of total fluence versus imaging depth at varying distances. (**f**) Plot of total fluence at the surface of placenta (depth, *z* = 17 mm) versus distance. The shading in the plots (**b**, **e**) from left to right represents a water coupling layer on the left-hand side followed by the tissue layers: skin, subcutaneous adipose tissue, abdominal muscle, and placenta respectively.

The simulated fluence distribution at varying incident angles is shown in Fig. 2(b). The plotted fluence is the total fluence calculated by summing the fluence of each voxel within each 23 mm $\times$ 1 mm (*x* − *y*) axial imaging plane along the z axis. Light directed towards the skin surface at smaller incident angles was less absorbed by the superficial layers, i.e. skin and adipose tissue, in comparison to light at larger incident angles. In Fig. 2(c), the light fluence at the surface of the placental tissue was plotted versus varying incident angle. Based on our simulations, a 20° incident angle provides the highest fluence at the surface of the placenta, which within our multilayer tissue model is 17 mm below the skin surface.

The total fluence of each *x* − *y* imaging plane versus distance within the tissue is shown in Fig. 2(e). As predicted, closer to the skin surface, the fluence is high, but fluence quickly decreases with increasing tissue depth. The fluence at the surface of the placenta was plotted versus distance in Fig. 2(f). We found that a 10 mm distance between the bifurcated light sources resulted in the highest fluence at the placental surface, 17 mm below the skin surface.

**Figure 3:**
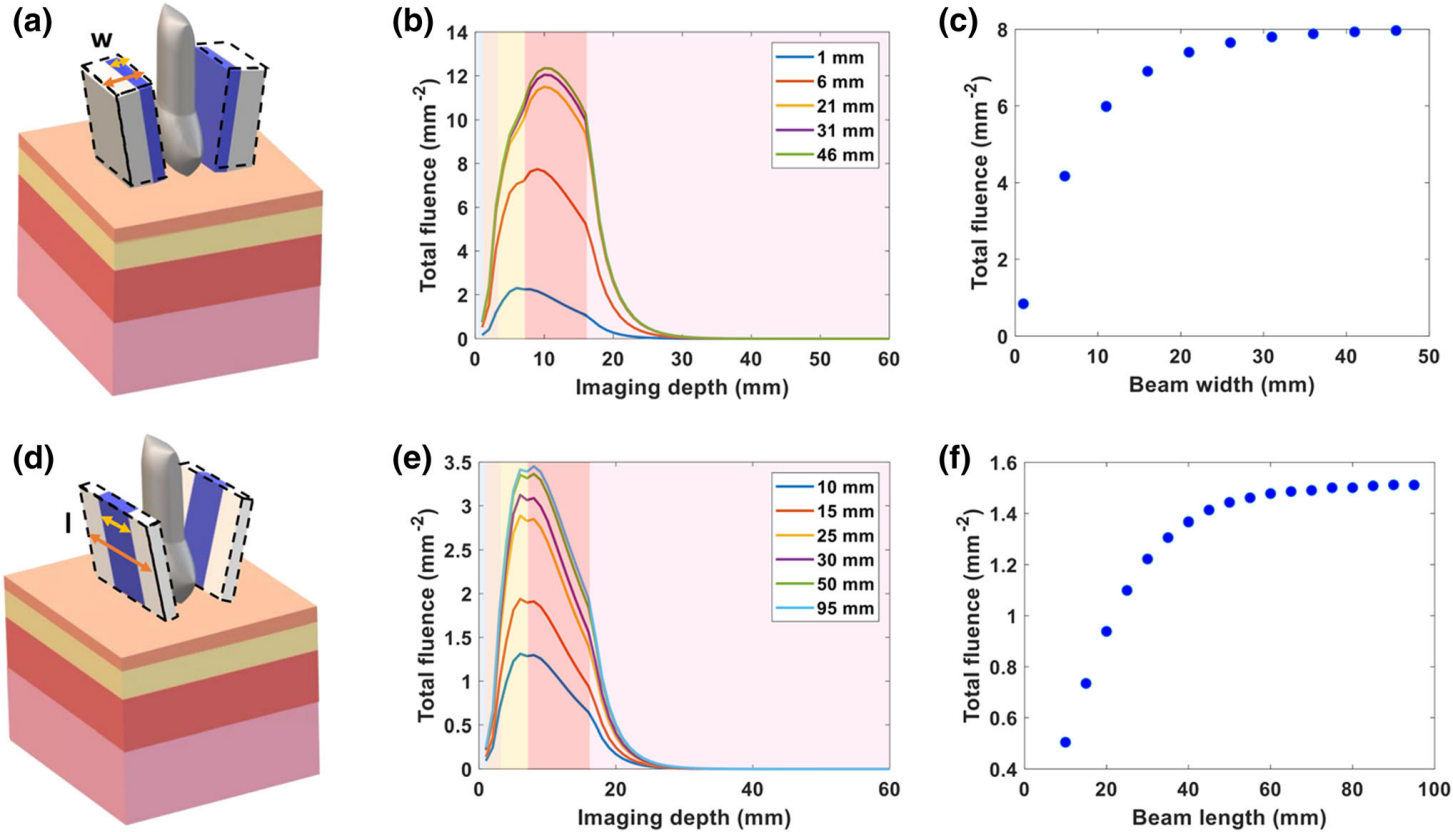
Increasing beam width and beam length improve delivery of 808 nm light. (**a**) Schematic showing variation in beam width along the elevational axis of the transducer. (**b**) Plot of total fluence versus imaging depth at varying laser beam widths. (**c**) Plot of total fluence at the surface of the placenta (depth *z* = 17 mm) versus beam width. (**d**) Schematic showing variation in beam length along the lateral axis of transducer. (**e**) Plot of total fluence versus imaging depth at varying laser beam lengths. (**f**) Plot of total fluence at the surface of placenta (depth *z* = 17 mm) versus beam length. The shading in the plots (**b**, **e**) from left to right represents water and the tissue layers of skin, subcutaneous adipose tissue, abdominal muscle, and placenta respectively.

The impact of beam width on the total fluence of the *x* − *y* imaging plane versus imaging depth is shown in Fig. 3(b). As the width of the light source is increased, the achievable fluence at increasing depth also increases. As the width increases beyond the width of the imaging plane, this affects plateaus. In Fig. 3(c), the fluence at the surface of the placenta is plotted versus beam width. Light delivered with a 23 mm width beam increased the fluence at the surface of the placenta 9× in comparison to the 1.25 mm beam width provided by the commercial LZ250 bifurcated fiber. The fluence values plateau for beam widths beyond 23 mm. Varying the beam width had a greater impact on the fluence at the placental surface, in comparison to the incident angle and distance between the bifurcated fibers. The total fluence at each *x* − *y* imaging plane versus imaging depth, while varying beam length, is shown in Fig. 3(e). The fluence plateaus as beam length approaches the lateral length of the transducer. The fluence delivered to the surface of the placenta is plotted versus beam length in Fig. 3(f). The fluence at the placental surface (*z* = 17 mm) increased 2× as the beam length increased from 10 to 25 mm.

**Figure 4:**
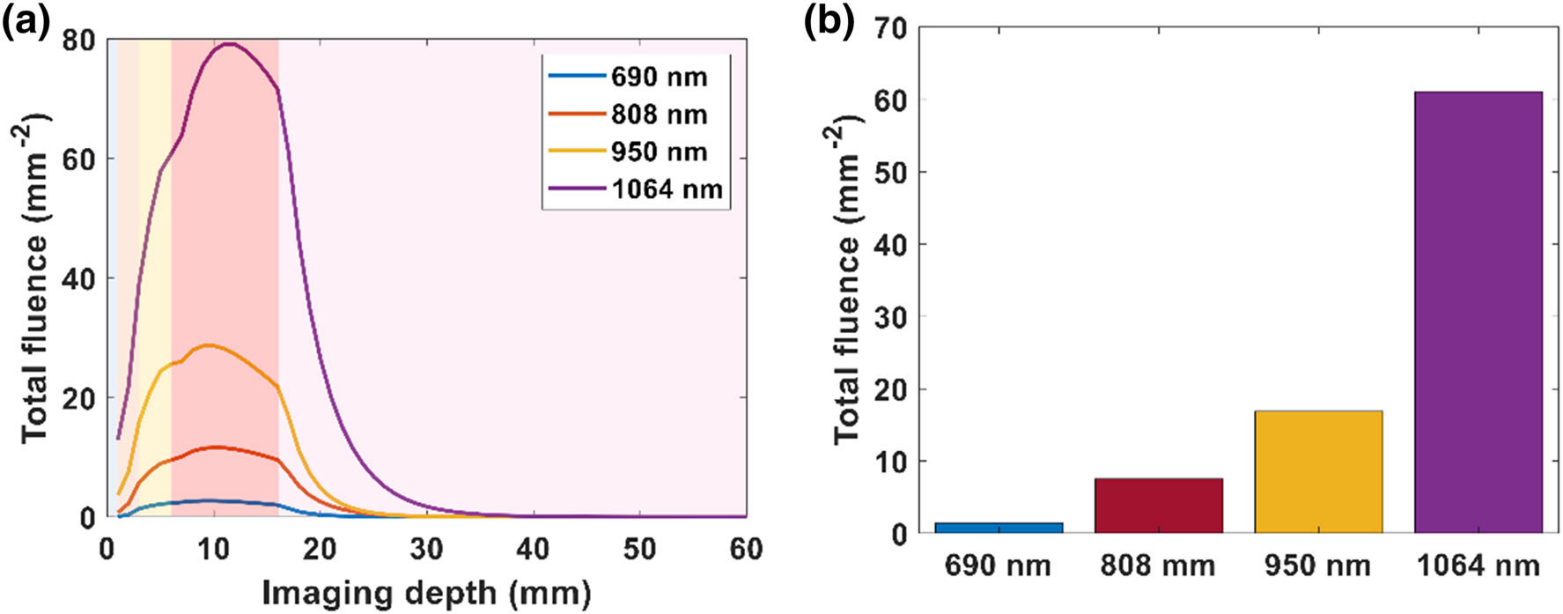
(**a**) Plot of total fluence versus imaging depth at varying wavelengths. (**b**) Bar plot of total fluence at the surface of placenta (depth *z* = 17 mm) at different wavelengths. The shading in the plot (**a**) from left to right represents water followed by the layers of skin, subcutaneous adipose tissue, abdominal muscle, and placenta respectively.

Based on the simulations, we selected the optimized geometric parameters of an incident angle 20°, distance 10 mm, beam width and beam length of 23 mm, and simulated varying wavelengths. We scaled the input energy for each wavelength using Eq. (3) to maintain the input fluence at the wavelength-dependent MPE limit. The fluence versus imaging depth at varying wavelengths is shown in Fig. 4(a). $R_{mpe}$, the ratio of the MPE limit of a specific wavelength to the MPE limit of 1064 nm wavelength laser pulses for 690, 808, 950 and 1064 nm were 0.2, 0.33, 0.63, and 1, respectively. Of the selected wavelengths, 1064 nm provided the highest fluence distribution in the multilayer tissue model across the tissue. Fluence at the surface of placenta (*z* = 17 mm), as shown in Fig. 4(b), indicates that 1064 nm light provides 8× the fluence of the 808 nm light. We also implemented our optimized light delivery system to observe how the percentage of melanin affects the light delivery depth for different skin types, as shown in the supplementary material (Sect. S6).

**Figure 5:**
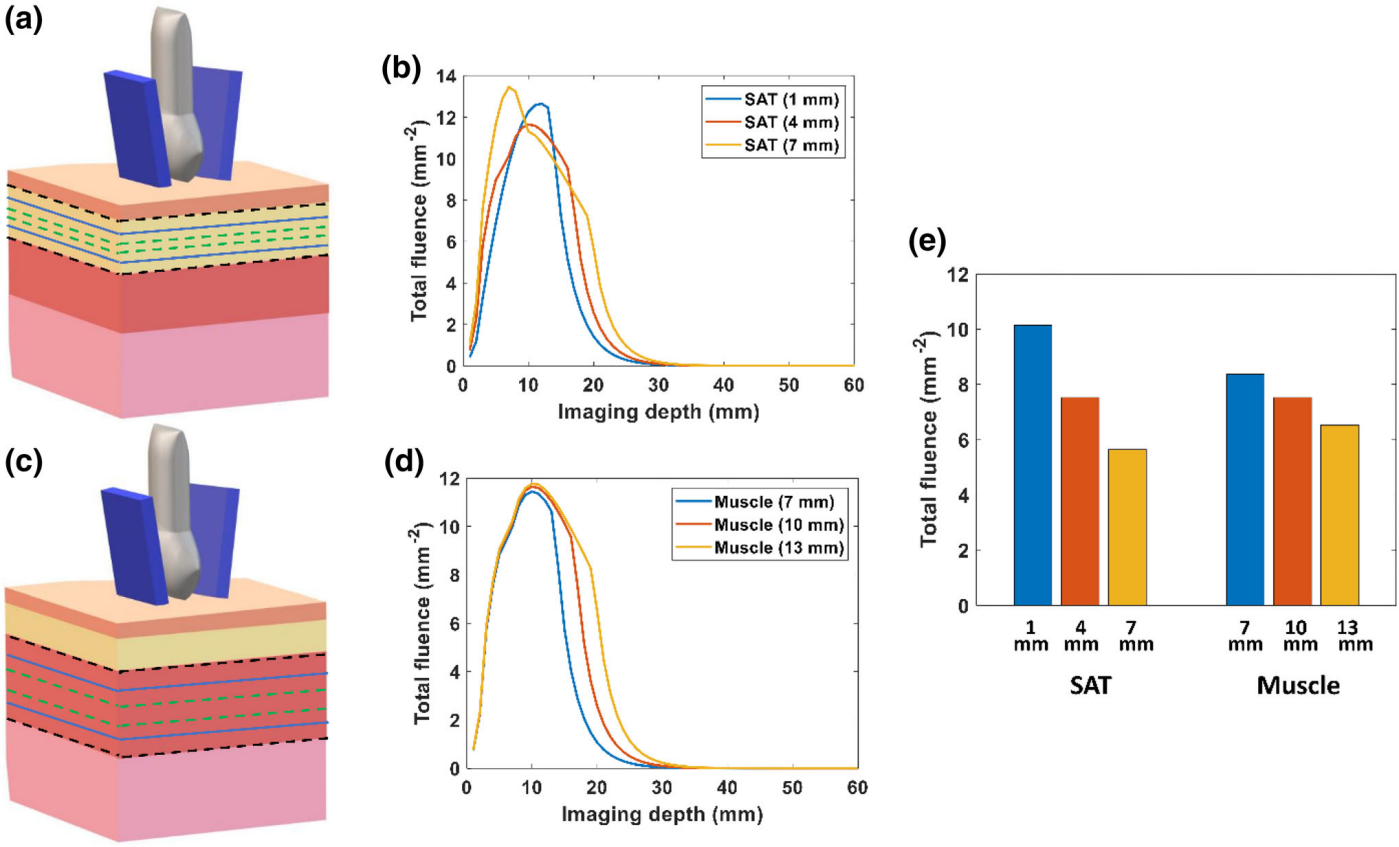
Effect of tissue thickness on fluence distribution in the multilayer tissue model with delivery of 808 nm light. (**a**) Variation of subcutaneous adipose tissue (SAT) thickness between 1 and 7 mm. (**b**) Plot of total fluence versus imaging depth at varying thicknesses of adipose tissue layer. (**c**) Variation of abdominal muscle thickness between 7 and 13 mm. (**d**) Plot of total fluence versus imaging depth at varying thickness of abdominal muscle layer. (**e**) Bar plot of the total fluence at the surface of placenta (*z* = 17 mm) with varying tissue thicknesses of adipose tissue and muscle layers.

Figures 5(b) and 5(d) show the simulated fluence distribution versus imaging depth with varying adipose tissue and abdominal muscle tissue thickness. As expected, a thinner layer of adipose tissue allows for the delivery of light deeper into the tissue, as shown in Fig. 5(b). Thicker adipose tissue absorbs more light, but the fluence distribution was similar in the tissue below the adipose layer. As shown in Fig. 5(d), the abdominal muscle thickness does not greatly affect the fluence distribution in the multilayer tissue model. Figure 5(e) displays a bar graph of the fluence at the surface of the placenta (*z* = 17 mm) with varying thicknesses of adipose tissue and muscle.

### *Ex Vivo* Tissue Imaging Experiment

**Figure 6:**
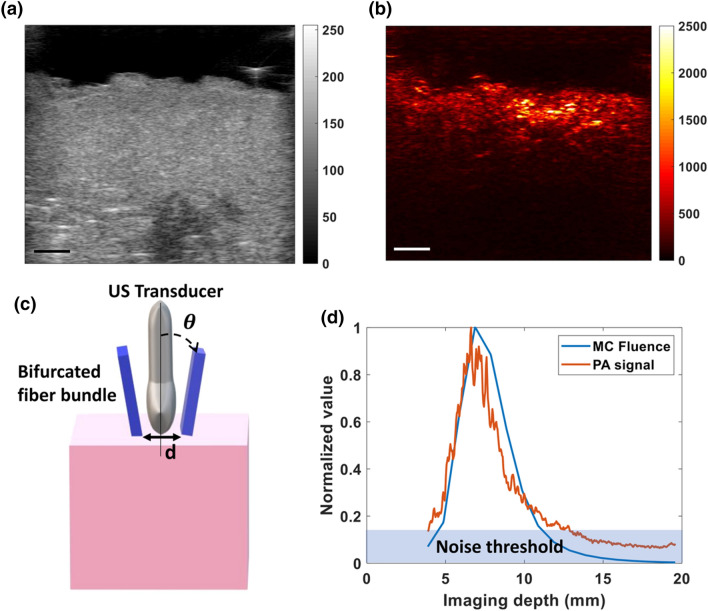
(**a**) B mode ultrasound image of a single cotyledon of a term human placenta, (**b**) photoacoustic image of a placenta at 808 nm, (**c**) Schematic of experimental set up of *ex vivo* imaging of a cotyledon of placenta. *θ* indicating incident angle of laser beam and *d* is the distance between bifurcated light source. (*d*) Plot of the normalized photoacoustic signal of a single cotyledon and Monte Carlo-simulated total fluence versus imaging depth. The blue region in the graph delineates the noise threshold, indicating photoacoustic signal generation as deep as 1.3 cm within the placental tissue. The scale bar is 3 mm and the total imaging depth is 20 mm.

We performed photoacoustic imaging of *ex vivo* human placental tissue and compared these image results to a matched simulation. Figures 6(a) and 6(b) show the B mode image and the 808 nm photoacoustic image acquired of a single cotyledon of an *ex vivo* human placenta. Figure 6(d) shows that our simulated fluence distribution as a function of depth is similar to that of the actual photoacoustic signal generated by the placenta. The depth at which a minimum photoacoustic signal could be detected in the *ex vivo* placenta tissue was calculated to be 1.29±0.02 cm for three placentas with three cotyledons each.

**Figure 7:**
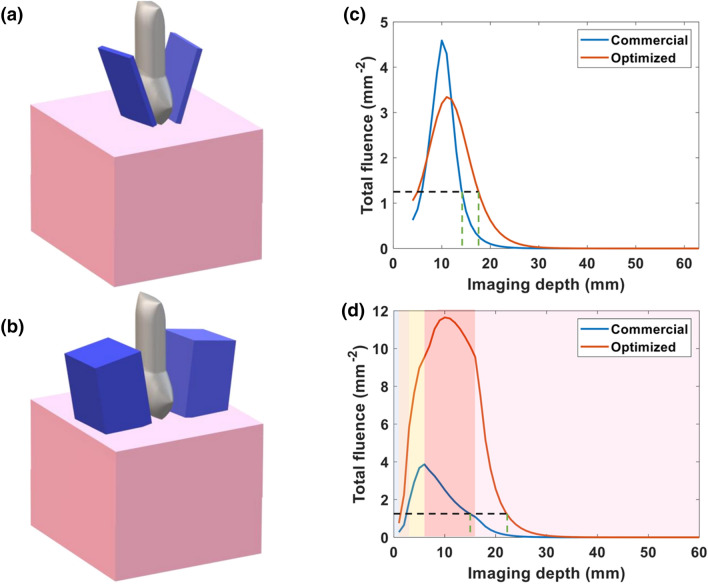
(**a**) Geometry of commercial light delivery design, (**b**) Geometry of optimized light delivery design. (**c**) Plot of total fluence of commercial and optimized light delivery versus imaging depth for a single layer of placenta at 808 nm. (**d**) Plot of total fluence of commercial and optimized light delivery versus imaging depth for multilayer tissue at 808 nm. The black dashed line indicates the threshold fluence value necessary to generate photoacoustic signal. The corresponding imaging depth for commercial and optimized light delivery systems is indicated with green dashed lines (1.3 and 2.16 cm respectively). The shading in the plot (**d**) from left to right represents water and the layers of skin, subcutaneous adipose tissue, abdominal muscle, and placenta respectively.

Next, we used simulations to compare our optimized light delivery geometry to the built-in fiber bundle of the LZ250 transducer. Figures 7(a) and 7(b) show the geometry of the built-in fiber bundle and our optimized light delivery, respectively. We plotted the fluence versus imaging depth of the built-in fiber bundle and optimized light delivery in Fig. 7(c). Using the same background photoacoustic signal threshold as above, we determined through this simulation that an optimized light delivery fiber bundle would increase the photoacoustic imaging depth within the placental tissue itself from 1.3 cm (with conventional commercial bifurcated bundle) to 1.8 cm (with our optimized light delivery). A more realistic simulation, with the multilayer tissue model to represent skin, adipose tissue, and muscle, shows that optimization of the light delivery improves imaging depth from 1.3 to 2.16 cm (Fig. 7(d)). The simulation variation was less than 0.001. Since the placenta tissue is estimated to be 1.7 cm beneath the skin, this improvement in imaging depth indicates the feasibility of human transabdominal photoacoustic imaging of placental tissue.

## Discussion

We have investigated the impact of the light delivery geometry and wavelength on photoacoustic imaging depth, towards the goal of enabling transabdominal imaging of the placenta. A Monte Carlo simulation was implemented to simulate the light delivery to a multilayer tissue model of the abdomen. In optimizing the light delivery geometry, we found that at constant fluence, the area of the beam has a greater impact on fluence delivery than incident angle and the distance between bifurcated light source. It has been previously established that increasing beam diameter increases the depth of light delivery.^3^,^25^ However, the simulated tissue depth was relatively shallow (less than 0.5 cm). Our simulation results, using a more complex multilayer abdominal tissue model and simulating a deeper tissue volume, demonstrate that a higher fluence can be achieved within deeper tissue (> 1.7 cm) by increasing the beam area. In prior studies, the maximum beam width as a function of imaging depth simulated plateaued around 0.4–1 cm for 0.15–0.5 cm imaging depth.^3^,^25^ However, in our simulation, which studied deeper tissue and a more complex tissue model of the abdomen, the optimal beam width and length plateaued at 2.3 cm. Since this plateau is limited by the imaging depth, as well as the imaging window (axial plane) of the transducer, a wider imaging field of view would likely benefit from a proportionately wider beam. The light wavelength also affects the fluence distribution within the tissue layers. Longer wavelengths, in the NIR II window, allow higher fluence to be delivered to the placental tissue, in comparison to wavelengths in the NIR I window. Our analysis of the effect of tissue thickness shows that thicker adipose tissue does reduce light delivery slightly to the placenta. However, the thicker adipose and muscle tissue do not have as large of an impact as the laser light geometry and wavelength. We validated our Monte Carlo simulation method by comparing the simulated results with a photoacoustic image of *ex vivo* placental tissue, to show that our optimized light delivery geometry improves imaging depth within the *ex vivo* placenta by 1.6× in comparison to the commercial light delivery system.

To mimic abdominal tissue in our simulations, skin, subcutaneous adipose tissue, abdominal muscle, and placenta were modeled. The absorption and scattering coefficients were calculated for all tissue layers for these simulations, rather than utilizing measured values from literature.^23^ We found that values of real tissue reported in the literature had discrepancies and missing data which led to our choice to use calculated values, as these were more consistent and reliable. Since the optical properties of the placenta have not been tested and reported, we simulated the placenta using the optical properties of the liver, since it is similar in blood volume to the placenta. The placenta has three distinct layers: the basal plate, intervillous space, and chorionic plate.^19^ Though these layers have different structures, hemoglobin is the dominant light-absorbing chromophore within all layers. Confirming this assumption, we found in preliminary experiments that the spectrum of the PA signal did not vary with depth or the placental layer in our *ex vivo* imaging experiments, justifying our choice to treat the placenta as a homogenous tissue whose optical properties are dominated by hemoglobin. Our calculation of optical properties of skin likely overestimates the impact of melanin shown in the supplementary (Sect. S6). Given preeclampsia disparately affects Black and Hispanic women, future model refinement will focus on the effect of higher melanin content on fluence distribution.

The light delivery geometry has a large impact on the fluence delivered to the placenta. Light at smaller incident angles with respect to the transducer achieves higher fluence at the placental surface, however with an exception for light at a 0° incident angle. As Fig. 2(c) shows, a slight angular incidence of light focuses the light beam at deeper region and allows light to propagate further in tissue. As the incident angle increases beyond 20°, the higher fluence is absorbed by the superficial layers such as the skin and adipose tissue. Since the photons transition from a ballistic regime to a diffuse regime within these superficial layers, the fluence decays rapidly with depth at these larger incident angles. A smaller distance between bifurcated light source also improves fluence distribution at depth, as light is delivered closer to the imaging plane of the transducer. In practice, the transducer itself will likely limit how closely a bifurcated fiber could approach the imaging plane.

Though a smaller incident angle and distance can improve fluence deposition at the surface of simulated placenta, the impact of these parameters on fluence is not remarkable in comparison to the width and length of the light beam. When we increased the beam width and length along the elevational and lateral axis of transducer to 23 × 23 mm^2^, we found a 9× increase in fluence at the surface of the simulated placenta, in comparison to the light delivery of the commercial system in our simulation studies. The optimal size of the beam width and length will depend on the imaging width along lateral axis of transducer, since this fluence increase plateaus after exceeding the imaging plane width. Finally, the effect of wavelength on fluence distribution was studied. As wavelengths in NIR II have higher MPE limits, higher fluence can be delivered to the tissue at these longer wavelengths. In our study, 1064 nm shows 8× greater fluence deposition at the placental surface in comparison to an 808 nm wavelength. In our simulations, we have chosen to use MPE limits to define our simulated fluence, rather than assuming light delivery is limited to a finite laser energy. While our in-house laser is limited to a maximum 80 mJ, and cannot experimentally achieve the fluences simulated here, nanosecond pulsed lasers providing up to 500 mJ are commercially available and would allow the simulations shown here to be realized experimentally. We anticipate continued advances in laser manufacturing design to achieve the MPE limits for these larger area beams.

We used a multilayer tissue model of the abdomen in simulations to study the effect of tissue thickness on the fluence distribution of the optimized light delivery geometry. In pregnancy, as in all humans, it would be normal for these layers to vary with obesity and anatomy. Additionally, adipose tissue thickness is a known barrier to ultrasound image quality, so we were particularly interested in determining the impact of adipose tissue on light delivery. Through our simulations, we found that the subcutaneous adipose tissue has a greater impact on the fluence distribution than the abdominal muscle. Since the adipose tissue layer is superficial and highly scatters photons, a thinner adipose tissue does achieve higher fluence at the surface of the placenta. The abdominal muscle is within the diffuse regime of light in our simulation, and therefore varying the thickness of this layer does not greatly impact the fluence distribution at the placental surface.

To determine the maximum imaging depth likely to be achieved with our optimized light delivery geometry, we compared the simulated fluence distribution of the optimized system with the fluence distribution of a commercial system. With the commercial photoacoustic transducer, we were able to generate photoacoustic signal 1.3 cm within the highly absorbing placental tissue. With our optimized light delivery system, we are able to improve this imaging depth to 1.8 cm in simulations. When we simulated the commercial transducer with the multilayer tissue model, we found that the minimum detectable photoacoustic signal was above the placental layer (1.3 cm depth in the model). However, our simulated optimized light delivery system was able to deliver light through the skin, adipose, and muscle, to reach the placenta (2.16 cm depth in the model). Though our simulated optimized light delivery improves imaging depth from 1.3 to 2.16 cm, the simulations-predicted improvement in imaging depth needs to be validated experimentally. Our noise threshold selection may underestimate the achievable imaging depth, since the gel coupling layer we used to determine our imaging noise threshold is highly affected by reflection artifacts. Additionally, alternative noise removal techniques may further increase the achievable imaging depth of our optimized light delivery.

For the *ex vivo* imaging experiments presented here, we used a high frequency transducer to acquire high resolution PA images. Since this high frequency transducer is limited by imaging depth, a lower frequency (1–5 MHz) transducer would be used for clinical translation of transabdominal photoacoustic imaging, typically achieving a US imaging depth up to 10 cm. Safety is also a concern for the clinical translation of *in vivo* imaging of the placenta. We have scaled our light source to the maximum permissible exposure limit ratio of laser pulse for each wavelength following ANSI Z136 guidelines.^2^ Since nanosecond pulsed lasers with low repetition rates are typically used to generate photoacoustic signal, the thermal effects are negligible, with an expected local temperature rise of less than 0.1 degrees.^6^ Mechanical effects like photodisruption can occur for nanosecond pulse above 100 J/cm^2^ fluence,^36^ which is much higher than our proposed optimized light delivery and also not likely to cause a safety concern based on current theory and predictions.

In conclusion, this comprehensive analysis of the impact of light delivery geometry and wavelength has identified methods to improve fluence delivery for photoacoustic imaging of placental tissue. Our simulations demonstrate that the beam area plays the largest role in fluence distribution to deeper regions, in comparison to the light angle of incidence and distance between bifurcated light sources. The longer wavelengths of the NIR-II allow the implementation of a higher MPE limit, and higher fluence delivery to deep tissues, despite the NIR I window being more commonly used to generate photoacoustic images. Additionally, we found through our simulations that the variation in the tissue thickness of the superficial layers does not greatly impact the available fluence at placental surface. Our simulated placental tissue study shows that an optimized light delivery design could be capable of generating photoacoustic signal from the transabdominal view of the placenta, with a 1.6× improvement in imaging depth in comparison to a conventional design. Using these simulations to guide improvements in the light delivery design, photoacoustic imaging may be likely to achieve *in vivo* functional monitoring of the human placenta.

## Electronic supplementary material

Below is the link to the electronic supplementary material.

Supplementary material 1 (DOCX 174 kb)
