# Supplementary material for: Towards Transabdominal Functional Photoacoustic Imaging of the Placenta: Improvement in Imaging Depth Through Optimization of Light Delivery
Source: Ann Biomed Eng. Author manuscript; Available in PMC 2022 Feb 1. (PMC8373763; doi:10.1007/s10439-021-02777-0)
Supplement: Electronic Supplementary [file NIHMS1712723-supplement-Electronic_Supplementary.docx]

**S1. MCXLAB- Monte Carlo Simulation:**

In the experimental simulation, photons were launched from the light source towards the tissue volume. Within the tissue volume, the optical properties of the absorption coefficient, scattering coefficient, anisotropy factor, and refractive index were defined for each voxel of size 1 mm^3^. In the simulation, water was used to interface the light source and the tissue, since photoacoustic imaging typically uses an aqueous acoustic coupling layer between the imaging transducer and the tissue. The photon propagation was initiated for 5 ns, matching a typical laser pulse width used to generate photoacoustic images. The simulation was performed with boundary conditions set to be non-reflective of light. The output of the Monte Carlo simulation was the normalized light fluence distribution in the 3D volume resulting from a unitary light source. The simulation was executed using an Intel Xeon Gold 6134 3.2GHz, 3.7GHz processor with a NVIDIA® Quadro® P4000, 8GB graphics processing unit. The simulation speed was 14243.32 photon/ms.

**S2. Calculation optical properties of multilayer tissue model:**

To calculate the reduced scattering coefficient $\mu_{s}^{'}$, we used the following equation, ^4^

$$\mu_{s}^{'}={a\left( \frac{\lambda}{500 nm} \right)}^{-b} (1)$$

where, $\lambda$ is the wavelength of light source normalized by reference wavelength 500 nm to yield a dimensionless value,$a$ is the value of $\mu_{s}^{'}$ at 500 nm wavelength, and $b$ is the scattering power. The calculated values of the reduced scattering coefficient of each tissue are provided in Table 1. Next, we calculated the absorption coefficient $\mu_{a}$ using the equation, ^4^

$$\mu_{a}=BS\mu_{a.oxy}+B\left( 1-S \right)\mu_{a.deoxy}+W\mu_{a.water}+M\mu_{a,melanosome} (2)$$

where B is the average blood volume fraction, S is the oxygen saturation of mixed arterio-venous vasculature,$\mu_{a.oxy}$ is the wavelength-dependent absorption coefficient of oxyhemoglobin,$\mu_{a.deoxy}$ is the wavelength-dependent absorption coefficient of hemoglobin, W is the water content of tissue, $\mu_{a.water}$ is the wavelength-dependent absorption coefficient of water, M is the melanin volume fraction, and $\mu_{a,melanosome}$ is the wavelength-dependent absorption coefficient of melanin. For simulation, we used B values of 1.57%, 0.76%, 1.16% and 49.5% for skin, subcutaneous fat, muscle and liver of sheep respectively.^5^ We used an M value of 4.3% for skin, representing skin type 2,^1^ and zero for all other tissue layers. The values of $\mu_{a,melanosome}$ were calculated using the equation, $\mu_{a,melanosome}=1.7\times{10}^{12}\lambda^{-3.48} {cm}^{-1}$.^4^

**S3. Calculation of ratio of input energy:**

The ratio of input energy ($RE_{i}$) for each light source was scaled as follows,

$${RE}_{i}=N_{p}\times l\times w\times R_{mpe} (3)$$

where $N_{p}$ is the number of photons per voxel, ($l\times w)$ is the beam area in mm^2^ and $R_{mpe}$ is the ratio of MPE limit of a specific wavelength to the MPE limit of 1064 nm wavelength laser pulses (unitless). MCXLAB assumes a unitary light source with constant energy with units of number of photons.^3^ Using Equation 3, we calculated the ratio of input energy required to maintain a constant fluence at the surface of the skin, given the changing beam dimensions. When simulating the impact of varying wavelength, the ratio of input energy which maintains a fluence at the surface of the skin proportional to the MPE limits was calculated. We then scaled the simulation output results proportionately, assuming a linear response to the input energy. The resulting output is the fraction of photons absorbed per voxel of tissue at the imaging plane at a particular depth in 1/mm^2^ units.

**S4. Variation of light geometry parameters:**

In our simulation geometry, the incident angle ($\theta$) is defined as the angle between the light beam and the axis normal to the surface of the skin (the vertical axis of US transducer). The incident angle of the light beam was varied from 0° to 80° with a step size of 10° for simulation. The distance between bifurcated light source (10 mm) and beam area (23 mm x 1.25 mm) were kept constant while varying the incident angle. Each light beam was positioned on the surface of the multilayer tissue model at d/2 distance from the vertical axis of transducer. For simulation, the distance was varied from 10 mm to 23 mm. The other parameters, incident angle and beam area, were kept constant for all simulations while the distance was varied, with an incident angle of 20° and a beam area of 23 mm x 1.25 mm for this series of simulations.

The width ($w$) and length ($l$) of the light beam indicated the elevational and lateral axes of the transducer, respectively. We varied the beam width from 1.25 mm to 100 mm, maintaining the incident angle of 20°, distance between the bifurcated fiber bundles of 10 mm, and a beam length of 23 mm. Then we varied the beam length from 10 mm to 100 mm, maintaining the incident angle of 20°, distance between the bifurcated fiber bundles of 10 mm, and beam width 23 mm.

**S5. Ex vivo tissue imaging:**

**Placenta preparation and Photoacoustic imaging:**

Each placenta was washed with heparinized saline to clear excess blood and clots from the placenta. Three different cotyledons from three locations (central, medial and peripheral) were isolated from the placenta for photoacoustic imaging. Each cotyledon was placed on a transparent tray, securing the position using gelatin. Photoacoustic imaging of the cotyledon was performed using a Vevo 2100 (FUJIFILM VisualSonics, Inc., Toronto, Canada) with a LZ250 transducer with a central frequency of 20 MHz (axial and lateral resolutions of 75 μm and 165 μm respectively). For generation of the photoacoustic signal, an Opotek Phocus Benchtop laser (5-7 ns pulses, 10 Hz repetition rate; Opotek Inc., Carlsbad, CA, USA) was integrated with the Vevo 2100. The transducer has a built-in bifurcated fiber bundle for coupling the laser light to the imaged tissue. The dimensions of each light beam were 23 mm (beam width) × 1.25 mm (beam length) with an incident angle of 30°. The LZ250 transducer can image up to 3 cm deep; typically, a term placenta is about 2 to 4 cm thick at the center.^2^ We imaged the placenta with the LZ250 transducer as a compromise between achievable imaging depth and high spatial resolution to allow for anatomical identification. A B mode ultrasound image of the cotyledon was used to select a region of interest from a section with a relatively flat cotyledon surface. Then, photoacoustic images were acquired using laser light tuned to 808 nm, with a maximum fluence of 35 mJ/cm^2^ at the end of the fiber bundle.

**S6. The Effect of Melanin:**

We simulated three different skin types with different melanin concentrations for our optimized light delivery system at different wavelengths. For these simulations, we used melanin volume fractions of 2% (lightly pigmented skin), 12% (moderately pigmented skin), and 20% (highly pigmented skin). The simulation results shown in Figure S6 show the impact of varying concentration of melanin on light delivery in a four-layer tissue model of transabdominal imaging.


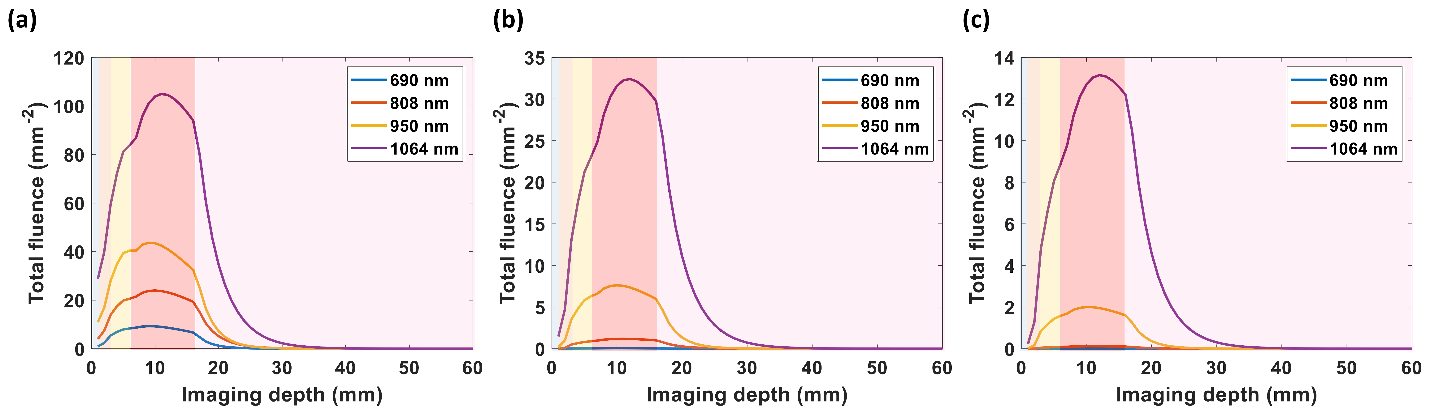


Figure S6: Plots of total fluence versus imaging depth at varying wavelengths for (a) lightly pigmented skin, (b) moderately pigmented skin and (c) highly pigmented skin.

Reference:

1. Ash C., M. Dubec, K. Donne and T. Bashford. Effect of wavelength and beam width on penetration in light-tissue interaction using computational methods. Lasers Med. Sci. 32: 1909-1918, 2017.

2. Fadl S., M. Moshiri, C. L. Fligner, D. S. Katz and M. Dighe. Placental Imaging: Normal Appearance with Review of Pathologic Findings. Radiographics 37: 979-998, 2017.

3. Fang Q. and D. A. Boas. Monte Carlo simulation of photon migration in 3D turbid media accelerated by graphics processing units. Opt. Express 17: 20178-20190, 2009.

4. Jacques S. L. Optical properties of biological tissues: a review. Phys. Med. Biol. 58: R37-61, 2013.

5. Weaver B. M., G. E. Staddon and M. R. Pearson. Tissue blood content in anaesthetised sheep and horses. Comp. Biochem. Physiol. A Comp. Physiol. 94: 401-404, 1989.
